# Efficacy Assessment of Montanide GEL 02 PR and Liposome‐Based Encapsulation as Adjuvants for an FrpA Subunit Vaccine Against Photobacteriosis in *Solea senegalensis*

**DOI:** 10.1111/jfd.70187

**Published:** 2026-04-15

**Authors:** Marta A. Lages, Miguel Balado, Abel M. Forero, Lucía Ageitos, Jaime Rodríguez, Carlos Jiménez, Manuel L. Lemos, Beatriz Magariños

**Affiliations:** ^1^ Department of Microbiology and Parasitology Aquatic One Health Research Center (ARCUS), Universidade de Santiago de Compostela Santiago de Compostela Spain; ^2^ Department of Aquaculture and Blue Biotechnology Centro Oceanográfico de Vigo (COV), Instituto Español de Oceanografía ‐ Consejo Superior de Investigaciones Científicas (IEO‐CSIC) Vigo Spain; ^3^ Department of Chemistry and CICA – Centro Interdisciplinar de Química e Bioloxía Universidade de A Coruña A Coruña Spain

**Keywords:** adjuvant, liposomes, Montanide GEL 02 PR, photobacteriosis, sole, vaccine

## Abstract

Photobacteriosis is a major threat to marine aquaculture, and the siderophore transporter FrpA represents a promising target for effective subunit vaccine development. In this study, we evaluated Montanide GEL 02 PR and phosphatidylcholine‐based liposomes as aqueous and safer alternatives to Freund's adjuvant for an FrpA‐based subunit vaccine in *Solea senegalensis* . Fish were immunized with recombinant FrpA formulated with either adjuvant, and induction of FrpA‐specific IgM antibody levels was measured. Vaccine efficacy was subsequently assessed by analysis of innate immune gene expression following early pathogen exposure and by survival analysis after infection challenge. Vaccination with rFrpA–Montanide GEL 02 PR induced the highest levels of specific IgM antibodies and conferred strong protection against photobacteriosis, achieving survival rates comparable to those reported for bacterin‐based vaccines. This formulation also promoted a rapid and coordinated up‐regulation of immune‐related genes, including transferrin (*tf*), lysozyme C1 (*lysC1*), and complement components C1q and C7. In contrast, rFrpA encapsulated in liposomes elicited weaker immune responses and limited protection. Overall, our results demonstrate that an FrpA‐based subunit vaccine formulated with Montanide GEL 02 PR represents a promising strategy for controlling photobacteriosis in aquaculture.

## 1 Introduction

Intensive aquaculture increases host–pathogen contact and promotes the emergence and persistence of infectious diseases (Kumar et al. 2024; Le Roux and Blokesch 2018). Conventional vaccines based on inactivated whole cells typically induce strong and long‐lasting immunity due to their intrinsic immunostimulatory properties (Ma et al. 2019); however, they may cause adverse effects, including toxicity, and offer limited formulation flexibility. Advances in vaccine design have enabled the development of subunit vaccines based on purified antigens, which provide improved safety and stability but often require adjuvant supplementation to achieve comparable efficacy (Ji et al. 2020; Ma et al. 2019).

*Photobacterium damselae* subsp. *piscicida* (*Pdp*) is the etiological agent of photobacteriosis, a severe disease affecting multiple marine fish species that causes major economic losses worldwide (Magariños et al. 1997; Toranzo et al. 2005). Virulence is associated with several factors, including a polysaccharide capsule (Magariños et al. 1996), AIP56 toxin (Freitas et al. 2025) and the siderophore piscibactin (Osorio et al. 2015). Iron acquisition systems are highly expressed during host infection, and their outer membrane transporters represent accessible and immunogenic vaccine targets (Lages et al. 2022, 2021). Currently available vaccines against photobacteriosis are mainly based on inactivated whole‐cell bacterins, frequently formulated with oil adjuvants and administered by injection (Andreoni and Magnani 2014; Weawsawang et al. 2024). Despite their protective efficacy, their associated adverse effects and limited flexibility highlight the need for alternative vaccine strategies (Miccoli et al. 2021). FrpA, the outer membrane transporter for piscibactin, has previously been validated as an effective subunit vaccine antigen when administered with Freund's adjuvant (Valderrama et al. 2019). Although Freund's adjuvant is widely used experimentally due to its strong immunostimulatory capacity (Munang'andu et al. 2020; Stills 2005; Tafalla et al. 2013), its severe side effects, such as granuloma formation, preclude commercial application (Stills 2005). This underscores the need for safer adjuvant alternatives suitable for aquaculture application (Tammas et al. 2024).

In this study, we evaluated the polymer‐based adjuvant Montanide GEL 02 PR (Seppic) and encapsulation in phosphatidylcholine‐based liposomes composed of 1‐palmitoyl‐2‐oleoyl‐*sn*‐glycero‐3‐phosphocholine (POPC) as aqueous‐based formulations and safer alternatives for the development of FrpA‐based vaccines. Vaccine efficacy in *Solea senegalensis* was assessed by analysing immune responses and survival following experimental vaccination and infection challenge.

## 2 Material and Methods

### 2.1 Recombinant FrpA (rFrpA) Expression and Purification

rFrpA was heterologously expressed in *Escherichia coli* BL21 (DE3) carrying the pET20b(+) C6His‐FrpA construct (Table 1). The construct was generated by amplifying the complete *frpA* gene from *P. damselae* subsp. *piscicida* DI21 strain using primers pET_FrpA_Fw and pET_FrpA_Rev (Table 2) and subsequently cloned into pET20b(+) via NcoI and XhoI restriction sites. Protein expression and purification were performed as previously described with minor modifications (Valderrama et al. 2019). Briefly, *E. coli* cultures were grown in LB‐Broth high salt (Sigma‐Aldrich, St. Louis, MO, USA) with 1 mM IPTG (Sigma‐Aldrich) at 28°C for 38 h. Cell pellets were resuspended (5 mL g^−1^ pellet) in lysis buffer (50 mM Tris–HCl pH 8.9, 500 mM NaCl, 10 mM imidazole, lysozyme, DNase I, and protease inhibitors) and incubated for 10 min at 4°C. Cells were lysed by sonication (five cycles, 40 s on/2 min off), followed by solubilization with 1% sodium N‐lauryl sarcosinate and 1% Elugent (Merck, Burlington, MA, USA). After centrifugation at 6000 rpm/4°C for 20 min, the supernatant was ultracentrifuged (120,000 rpm/4°C for 15 min), filtered (0.45 and 0.22 μm), and purified by Ni^2+^ affinity chromatography using an NGC Discover 10 Chromatography System (Bio‐Rad, Hercules, CA, USA) and a 5 mL HisTrap column (Cytiva, Marlborough, MA, USA). Protein‐containing fractions were concentrated and buffer‐exchanged using Amicon Ultra 50 K devices (Merck). Protein concentration was determined using a Qubit fluorometric quantitation system (Thermo Fisher Scientific, Waltham, USA).

**TABLE 1:** Strains and plasmids used in this word.

Strain or plasmid	Description	Reference/source
Strain		
*Photobacterium damselae* subsp. *piscicida* (*Pdp*)		
DI21	Highly virulent strain isolated from gilthead sea bream ( *Sparus aurata* ) in 1990, Spain	Laboratory collection
*E. coli*		
BL21	F–*ompT gal dcm lon hsdSB* (rB– mB–) [*malB+*]K‐12 (λS)	Laboratory stock
Plasmid		
pET20b (+) C6His‐FrpA	pET20b(+) expression vector with *frpA*	This study

**TABLE 2:** Oligonucleotide primers used for *frpA* amplification and RT‐qPCR immune response evaluation (described by Núñez‐Díaz et al. 2017).

Oligonucleotide (5′ → 3′)	
*frpA*	
pET_FrpA_Fw	CGGCCATGTACAGGAACAGTTTCTCGCTCTCTCC
pET_FrpA_Rev	CCCTCGAGCCACTCTAGCTTCATATTCACACCC
Beta‐actin 2	
Beta‐actin 2_F	AATCGTGACCTCTGCTTCCCCCTGT
Beta‐actin 2_R	TCTGGCACCCCATGTTACCCCATC
Complement C1q	
C1q_F	CTACGCTTCTAACAGTGTGATCCT
C1q_R	AGCTGCACACACACCTCATC
Complement C7	
C7_F	GGCACACACTATCTGTCGCAGGGCTC
C7_R	GGCGAACGCCTGATGGTTTAACTCCAG
Lysozyme type C1	
LysC1_F	CAGATCAACAGCCGCTATTGG
LysC1_R	GCTGATTCCACATGCATTTGAAGTG
Transferrin	
TF_F	CGTTGTATCGCTCCTCATCTCTGGGTTTG
TF_R	CTACTTCAGTGAGAGCTGTGCCCCTGGAG

### 2.2 Verification of Purified rFrpA Integrity by SDS‐PAGE and Western Blotting

Fractions containing rFrpA obtained after purification were analysed by sodium dodecyl‐sulfate polyacrylamide gel electroforesis (SDS‐PAGE) and Western blotting. Proteins were separated by SDS‐PAGE (10% polyacrylamide gel) and transferred onto Immun‐Blot polyvinylidene difluoride (PVDF) membrane (Bio‐Rad) using a Mini Trans‐Blot Electrophoretic Transfer Cell (Bio‐Rad) according to the manufacturer's instructions. Membranes were blocked with 5% (w/v) skim milk (Sigma‐Aldrich) in PBS for 45 min at room temperature and washed with PBS containing 0.05% Tween 20 (Sigma‐Aldrich). rFrpA was detected using a 6 × His Tag mouse monoclonal antibody (His.H8, 1:1000 dilution) (Thermo Fisher Scientific), followed by HRP‐conjugated Goat anti‐Mouse secondary antibody (Thermo Fisher Scientific). Signal detection was performed using a chemiluminescent NZY Advanced ECL substrate (NZYTech, Lisbon, Portugal).

### 2.3 Liposomes Formulation and rFrpA Encapsulation

rFrpA was incorporated into liposomes using a hydration–extrusion method (Zhang 2023), with minor modifications (Figure S1). Lipid films were prepared from 1‐palmitoyl‐2‐oleoyl‐*sn*‐glycero‐3‐phosphocholine (5 mM) dissolved in chloroform. After solvent evaporation, the lipid film was hydrated with rFrpA (0.4 mg mL^−1^) in PBS (pH 7.4) and incubated for 1 h at 4°C to form proteoliposomes. Unilamellar vesicles were generated by sequential extrusion through 0.2 and 0.1 μm polycarbonate membranes (21 passes each) using a Mini‐Extruder (Avanti Polar Lipids, Alabaster, USA). Proteoliposomes were collected by ultracentrifugation (250,000 × *g*, 1 h, 4°C), resuspended in 2 mL PBS, and visualized by transmission electron microscopy (TEM) and scanning electron microscopy (SEM). Control liposomes were prepared using PBS alone. Protein encapsulation efficiency was indirectly quantified by measuring residual protein in the supernatant after ultracentrifugation using BCA protein assay kit (Abcam, Cambridge, UK). Finally, liposomes were adjusted to a protein concentration of 0.3 mg mL^−1^.

### 2.4 Preparation of rFrpA–GEL02 Vaccine Formulation

The rFrpA antigen solution in PBS was gently mixed with Montanide GEL 02 PR (Seppic, Paris, France) at a 10:1 (v/v) ratio, according to the manufacturer's instructions, to obtain the rFrpA–GEL02 formulation with a final protein concentration of 0.3 mg mL^−1^.

### 2.5 Fish Immunization Assay

*Solea senegalensis* fingerlings (mean weight ≈10 g) were acclimated for 15 days in 100 L tanks with recirculating filtered seawater, continuous aeration, and a constant temperature of 18°C. Fish were fed a commercial diet, and health status was monitored daily based on swimming behaviour, feeding activity, and external appearance.

**FIGURE 1:**
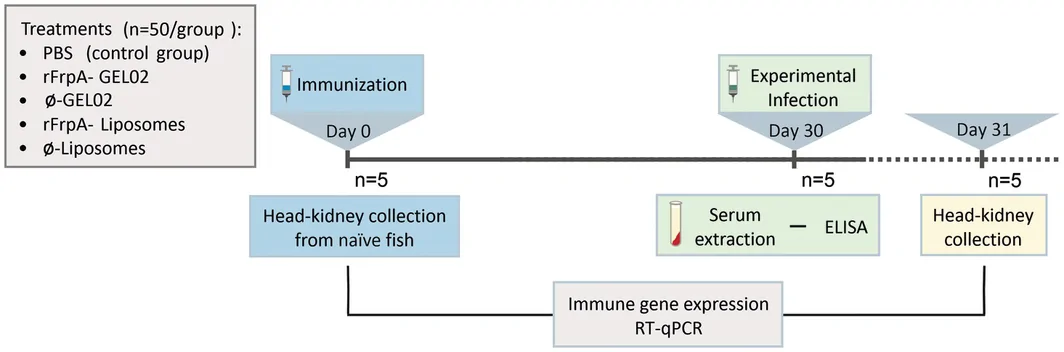
Experimental design of fish immunization and infection challenge. *Solea senegalensis* were randomly distributed into treatment groups (*n* = 50 fish per group) and intraperitoneally immunized on Day 0. Head kidney samples were collected from naïve fish prior to immunization (*n* = 5). Thirty days post‐immunization, fish were experimentally challenged with *P. damselae* subsp. *piscicida* DI21. Serum samples were collected for ELISA analysis (*n* = 5), and head kidney samples were collected 24 h (*n* = 5) post‐challenge for immune gene expression analysis by RT‐qPCR.

Prior to immunization, fish were randomly distributed into five groups (*n* = 50 per group). Each fish was intraperitoneally injected with 100 μL of one of the following formulations: (i) 30 μg rFrpA formulated with Montanide GEL 02 PR (rFrpA–GEL02); (ii) Montanide GEL 02 PR control (PBS:GEL02, 10:1; Ø–GEL02); (iii) 30 μg rFrpA encapsulated in liposomes (rFrpA–Liposomes); (iv) empty liposomes (Ø–Liposomes); or (v) PBS (PBS–Control) (Figure 1). Fish were monitored for 30 days post‐immunization.

### 2.6 Determination of Antibody Levels by Enzyme‐Linked Immunosorbent Assay (ELISA)

Antibody levels were determined 30 days post‐immunization. Blood samples were collected from the caudal vein of five randomly selected fish per group (Figure 1), allowed to clot for 2 h at 4°C, and centrifuged at 400 × *g*. Serum was recovered and stored at −20°C until analysis. FrpA‐specific IgM levels were quantified by indirect ELISA as previously published (Valderrama et al. 2019). Briefly, 96‐well microtiter plates were coated overnight at 4°C with 50 μL per well of rFrpA or PBS (control). Plates were washed with TTBS (0.5% Tween 20, 50 mM Tris–HCl, 150 mM NaCl, pH 7.5) and blocked with skim milk (Sigma‐Aldrich) in TBS for 1 h at 25°C. Serum samples diluted 1:1000 in PBS were added (50 μL per well) and incubated for 30 min at 25°C. Bound IgM was detected using a mouse anti–giant gourami IgM monoclonal antibody (1:1000; Aquatic Diagnostics Ltd., Stirling, UK), followed by horseradish peroxidase–conjugated goat anti‐mouse IgG (1:1000; Thermo Fisher). Signal development was performed using TMB substrate (Bio‐Rad) and stopped with 1 N H₂SO₄. Absorbance was measured at 450 nm using an iMark microplate reader (Bio‐Rad). All samples were analysed in triplicate, and results are expressed as mean values. Statistical differences between groups were evaluated using Student's *t*‐test.

### 2.7 Experimental Infection

Thirty days post‐immunization, fish were subjected to an experimental infection challenge (Figure 1). Fish were intraperitoneally injected with 100 μL of *P. damselae* subsp. *piscicida* strain DI21 at a dose of 3 × 10^5^ CFU mL^−1^ in PBS, corresponding to a dose previously reported to induce approximately 80% mortality in control fish (Valderrama et al. 2019). Fish were maintained at 19°C, and mortality was recorded daily for 10 days. *P. damselae* subsp. *piscicida* was re‐isolated from the internal organs of diseased fish to confirm infection. Vaccine efficacy was evaluated by survival analysis using Kaplan–Meier survival curves, and differences between groups were assessed using the log‐rank (Mantel–Cox) test. The relative percent survival (RPS) was calculated as: RPS = [1 − (% mortality in vaccinated group/% mortality in control group)] × 100.

All immunization and challenge procedures were conducted in accordance with European and Spanish regulations for the use of animals in scientific research (Directive 2010/63/EU) and were approved by the Bioethics Committee of the University of Santiago de Compostela (Protocol No. 15012/2022/001).

### 2.8 Tissue Sampling and Immune Response Analysis by RT‐qPCR

Real‐time quantitative PCR (RT‐qPCR) was used to analyse the expression of immune‐related genes in each fish group 24 h post‐challenge (Figure 1) (Núñez‐Díaz et al. 2017). Five fish per group were randomly selected, and head kidney tissues were collected and stored in TRIzol reagent (Invitrogen, Waltham, MA, USA) at 4°C.

Total RNA was extracted according to the manufacturer's instructions. RNA concentrations were determined using the Qubit RNA BR Assay Kit (Invitrogen), adjusted to 320 ng μL^−1^, and 4 μL of each sample were treated with DNase I (Thermo Scientific) to remove residual genomic DNA. Gene expression analysis was performed using the One‐step NZYSpeedy RT‐qPCR Green kit (NZYTech). Primers targeting β‐actin 2 (*actb2*, reference gene), transferrin (*tf*), complement component C1q (*c1q*), lysozyme C1 (*lysC1*), and complement component C7 (*c7*) are listed in Table 2. Reactions were performed in two technical replicates using the following cycling conditions: reverse transcription at 50°C for 20 min, polymerase activation at 95°C for 5 min, and 40 cycles of denaturation at 95°C for 5 s and annealing/extension at 60°C for 30 s. A melting curve analysis (65°C–95°C, 0.5°C increments every 5 s) was included to confirm amplification specificity. RT‐qPCR assays were carried out using a CFX96 Real‐Time PCR Detection System (Bio‐Rad). Relative gene expression levels were calculated using the 2^−ΔΔCt^ method, normalized to ACTB2. Gene expression data were analysed using one‐way ANOVA followed by Tukey's post hoc test for multiple comparisons, with *p* < 0.05 considered statistically significant.

## 3 Results

### 3.1 rFrpA‐GEL02 Formulation Induces the Production of FrpA Specific Antibodies in Fish Serum

Following rFrpA production, protein integrity and purification were confirmed by SDS‐PAGE and Western blot (Figure S2). Vaccine formulations (rFrpA–GEL02 and rFrpA–liposomes) and their corresponding controls were then prepared as described in Materials and Methods. Proteoliposome formation was visualized by TEM and SEM, showing spherical liposomes (Figure S3A,B).

**FIGURE 2:**
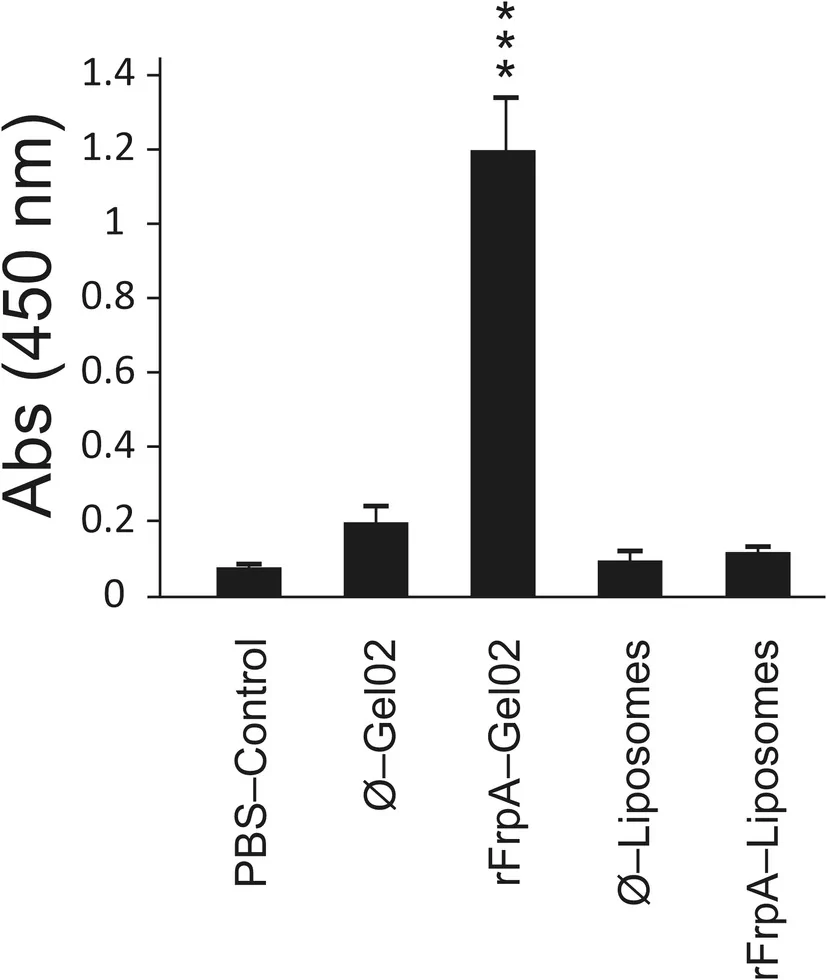
Anti‐FrpA IgM levels in fish sera determined by ELISA 30 days post‐immunization with rFrpA subunit vaccines formulated with Montanide GEL 02 PR (rFrpA–GEL02) or encapsulated in liposomes (rFrpA–Liposomes), compared with their respective controls: GEL02 alone (Ø–GEL02), empty liposomes (Ø–Liposomes), and PBS (PBS–Control). Data are expressed as mean ± SD. Asterisks indicate statistically significant differences as determined by Student's *t*‐test (*p* < 0.001).

Two groups of fish were immunized with rFrpA formulated with Montanide GEL 02 PR (rFrpA–GEL02) or encapsulated in POPC liposomes (rFrpA–Liposomes), together with their respective control groups treated with GEL02 alone (Ø–GEL02), empty liposomes (Ø–Liposomes), or PBS (PBS‐Control). Thirty days post‐immunization, anti‐FrpA IgM levels were quantified by ELISA (Figure 2). Fish immunized with rFrpA–GEL02 showed the highest antibody levels (*A*_450_ = 1.2), significantly higher than those of the other treatments. Fish of the rFrpA–Liposomes group showed lower antibody levels, reaching approximately 10% of those observed in rFrpA–GEL02. Control groups Ø–GEL02 and Ø–Liposomes showed minimal antibody levels.

### 3.2 rFrpA‐GEL02 Confers Protection Against Photobacteriosis

**FIGURE 3:**
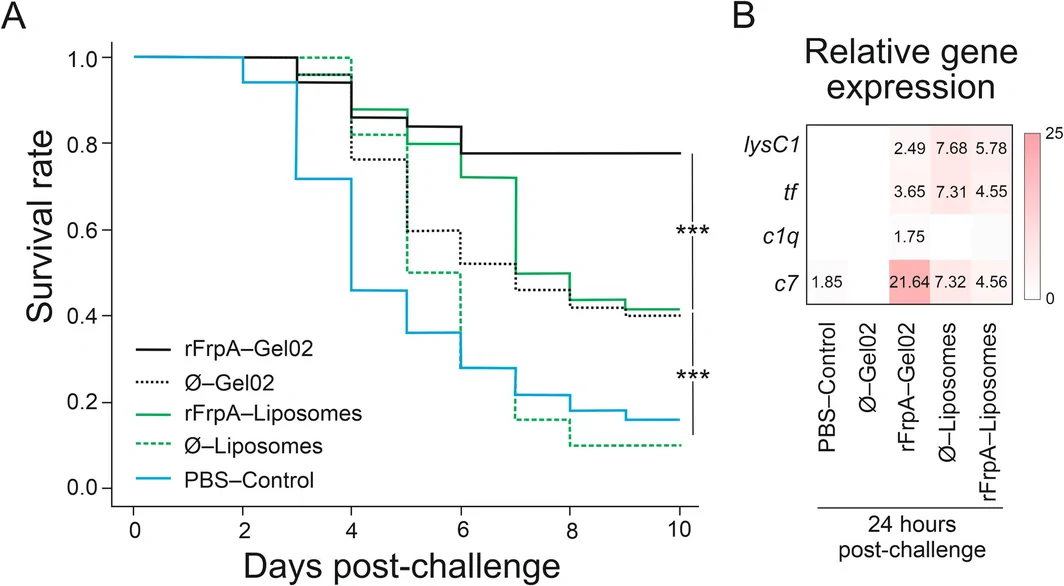
Protection and immune responses conferred by rFrpA subunit vaccines formulated with Montanide GEL 02 PR (rFrpA–GEL02) or encapsulated in liposomes (rFrpA–Liposomes), compared with their respective controls: GEL02 alone (Ø–GEL02), empty liposomes (Ø–Liposomes), and PBS (PBS–Control). (A) Survival curves of fish groups following intraperitoneal challenge with *P. damselae* subsp. *piscicida* (3 × 10^5^ CFU per fish). (B) Differential expression of immune‐related genes in the head kidney 24 h post‐challenge. The heatmap shows relative gene expression values calculated using the 2^−ΔΔCt^ method; only genes with fold‐change values ≥ 1.5 relative to naïve fish and showing statistically significant differences (*p* < 0.05) are displayed.

Thirty days post‐immunization, fish were challenged by intraperitoneal injection with *Pdp* strain DI21 (3 × 10^5^ CFU per fish), and mortality was monitored for 10 days (Figure 3A). Typical signs of photobacteriosis were observed in deceased fish, and *Pdp* was re‐isolated from internal organs. The PBS‐Control group showed rapid mortality starting at 2 days post‐challenge, reaching a final survival rate of 18%.

Among the vaccinated groups, rFrpA–GEL02 provided the highest level of protection, with a final survival rate of 78% (RPS = 73%), significantly higher than all control groups and the rFrpA–liposomes treatment, which reached a survival rate of 44% at the final of the experiment. No significant differences were observed between fish vaccinated with rFrpA–Liposomes and Gel02 control (Ø–GEL02), exhibiting intermediate survival rates ranging from 40% to 43% (RPS = 30%). In contrast, the Ø–Liposomes control group displayed a survival profile indistinguishable from that of the PBS control, with a final survival rate of 12.5%. These results suggest that rFrpA formulated with GEL02 confers protection against photobacteriosis when compared with the liposome‐based formulation.

### 3.3 rFrpA–Montanide GEL 02 PR Activates an Immune Response Following Pathogen Challenge

To determine the immune‐stimulatory capacity of FrpA‐based formulations, immune‐related gene expression was analysed in the head kidney of five fish per group 24 h after experimental challenge with *Pdp*. Fold‐change values relative to naïve fish (non‐treated and non‐challenged) are shown in Figure 3B. In the non‐vaccinated control group (PBS), only a weak induction of *c7* was detected (1.85‐fold), whereas *lysC1*, *tf*, and *c1q* were not significantly induced. Similarly, Ø–GEL02 control showed no significant induction of the immune genes analysed.

Fish vaccinated with rFrpA encapsulated in liposomes (rFrpA–Liposomes) exhibited increased expression of *lysC1* (5.78‐fold), *tf* (4.55‐fold), and *c7* (4.56‐fold), while *c1q* was not significantly induced. Likewise, fish treated with empty liposomes (Ø–Liposomes) showed induction of *lysC1* (7.68‐fold), *tf* (7.31‐fold), and *c7* (7.32‐fold), with no significant up‐regulation of *c1q*.

Notably, fish vaccinated with rFrpA–GEL02 displayed a marked up‐regulation of *lysC1* (2.49‐fold), *tf* (3.65‐fold), and especially *c7*, which reached a 21.64‐fold increase, whereas *c1q* showed only a weak induction (1.75‐fold).

## 4 Discussion

Vaccination is the most effective strategy for controlling fish diseases, reducing economic losses and limiting antibiotic use in aquaculture, thereby helping to prevent the spread of antibiotic resistance among bacterial species (Miccoli et al. 2021). Montanide GEL 02 PR is a polymeric adjuvant designed to improve the safety and efficacy of aqueous vaccines. Our results indicate that fish vaccinated with a single dose of rFrpA–GEL02 exhibited a rapid and robust immune response following pathogen exposure, consistent with high levels of FrpA‐specific IgM antibodies and showed protection against photobacteriosis, achieving a RPS of 73%. These values are comparable to those previously reported for rFrpA formulated with Freund's adjuvant after two immunizations and for the conventional *P. damselae* subsp. *piscicida* DI21 bacterin, which confer protection in the range of 70%–80% (Valderrama et al. 2019). Vaccine formulations including Montanide GEL 02 PR have been successfully used in vaccines against *Streptococcus agalactiae* in Nile tilapia (Wangkaghart et al. 2021), infectious pancreatic necrosis virus (IPNV)(Li et al. 2025), and infectious haematopoietic necrosis virus (IHN)(Lin et al. 2022) in rainbow trout, where it promoted strong and sustained immune responses without adverse effects (Li et al. 2025; Lin et al. 2022; Wangkaghart et al. 2021). The present work therefore provides the first evidence supporting the effectiveness of Montanide GEL 02 PR as an adjuvant in a subunit vaccine in aquaculture.

Synthetic liposomes composed of POPC constitute stable and safe delivery systems suitable for vaccine formulation (Mebarek et al. 2026; Negari et al. 2025). Although rFrpA–liposome–treated fish showed no detectable anti‐FrpA IgM production, post‐challenge up‐regulation of innate immune genes was observed, but this response was weaker than that induced by Montanide GEL 02 PR and did not translate into significant protection. This pattern suggests a non‐specific or suboptimal immune activation insufficient to control infection. Liposomes are widely used as antigen delivery systems due to their biodegradability, biocompatibility, and ability to protect antigens from degradation (Adams 2019; Fraziano 2018; Giddam et al. 2012; Kersten and Crommelin 2003), and liposome‐based vaccines have successfully protected fish against bacterial pathogens in previous studies (Harikrishnan et al. 2012; Irie et al. 2005). However, adjuvant efficacy strongly depends on lipid composition and surface charge, with cationic formulations generally inducing stronger immune responses (Lonez et al. 2012). The rFrpA–liposome formulation used here likely lacked optimal immunostimulatory properties, which may explain its reduced protective capacity.

Analysis of immune‐related gene expression further supported the efficacy of the rFrpA–Montanide GEL 02 PR formulation, as it induced the up‐regulation of transferrin, lysozyme C1, and the complement components C1q and C7. These genes have previously been associated with protective responses against *P. damselae* subsp. *piscicida* in sole (Núñez‐Díaz et al. 2017). Transferrin expression was strongly induced, supporting its role as a positive acute‐phase protein involved in iron sequestration and macrophage activation (Ali et al. 2023; Neves et al. 2009; Santos et al. 2022; Trites and Barreda 2017). Up‐regulation of lysozyme C1 likely contributed to bacterial clearance through cell wall degradation (Bayne and Gerwick 2001; Mokhtar et al. 2023). Activation of complement components C1q and C7 further indicates efficient engagement of antibody‐mediated recognition and membrane attack complex formation, key mechanisms in bacterial lysis (Dunkelberger and Song 2010). Collectively, these findings suggest that rFrpA–Montanide GEL 02 PR–vaccinated fish mounted a rapid and coordinated immune response, consistent with their enhanced survival. Importantly, previous studies suggest that Montanide GEL 02 PR is a safe adjuvant, showing no detectable tissue alterations in vaccinated fish (Lin et al. 2022; Wangkaghart et al. 2021). In line with these reports, no alterations in fish behaviour or macroscopic side effects, including inflammatory reactions, intestinal adhesions, granuloma, or discoloration at the injection site were observed during the present study.

In recent years, multiple vaccine strategies against photobacteriosis, including inactivated cells, OMVs, DNA vaccines, and subunit approaches, have been explored with variable efficacy (Lan et al. 2024; Pham et al. 2021; Ponce et al. 2021; Teixeira et al. 2023). Targeting FrpA offers a strategic advantage, as this virulence‐associated outer membrane transporter is essential for pathogenicity (Osorio et al. 2015). Its functional constraint reduces the likelihood of immune escape, supporting its use as a robust and stable antigen for next‐generation vaccines against photobacteriosis in aquaculture.

Overall, this study demonstrates that vaccination with the rFrpA–GEL02 formulation elicited a rapid and robust immune response following pathogen exposure, consistent with high levels of anti‐FrpA IgM antibodies and strong protective efficacy. Collectively, these findings demonstrate that an FrpA‐based subunit vaccine formulated with Montanide GEL 02 PR represents an effective strategy for controlling photobacteriosis in aquaculture.

## Author Contributions

**Marta A. Lages:** investigation, data curation, visualization, formal analysis, writing – original draft, writing – review and editing. **Miguel Balado:** conceptualization, investigation, visualization, formal analysis, writing – original draft, writing – review and editing, validation, supervision, methodology, data curation. **Abel M. Forero:** investigation, visualization, writing – review and editing. **Lucía Ageitos:** investigation, visualization, writing – review and editing. **Jaime Rodríguez:** investigation, supervision, formal analysis, writing – review and editing, funding acquisition. **Carlos Jiménez:** investigation, supervision, formal analysis, writing – review and editing, funding acquisition, project administration. **Manuel L. Lemos:** conceptualization, investigation, supervision, formal analysis, writing – review and editing, funding acquisition, project administration. **Beatriz Magariños:** conceptualization, investigation, supervision, formal analysis, writing – review and editing, funding acquisition.

## Funding

This work was supported by MCIN/AEI/10.13039/501100011033/FEDER (PDC2022‐133052‐C21/C22) and Xunta de Galicia (ED431C 2022/023, ED431C 2022/39, PRTR‐C17.I1).

## Conflicts of Interest

The authors declare no conflicts of interest.

## Supporting information


**Figure S1:** Schematic representation of rFrpA encapsulation into liposomes.
**Figure S2:** SDS‐PAGE and Western blot analysis of purified rFrpA following Ni^2+^ affinity chromatography.
**Figure S3:** Ultrastructural characterization of rFrpA‐loaded liposomes.

## Data Availability

The data that support the findings of this study are available on request from the corresponding author. The data are not publicly available due to privacy or ethical restrictions.
