# Supplementary material for: Efficacy Assessment of Montanide GEL 02 PR and Liposome‐Based Encapsulation as Adjuvants for an FrpA Subunit Vaccine Against Photobacteriosis in Solea senegalensis
Source: J Fish Dis. 2026 Apr 15;49(10):e70187. doi: 10.1111/jfd.70187 (PMC13532611; doi:10.1111/jfd.70187)
Supplement: Supplementary file 1 [file JFD-49-e70187-s001.docx]

**Supporting Information**

**Efficacy assessment of Montanide™ GEL 02 PR and liposome-based encapsulation as adjuvants for an FrpA subunit vaccine against photobacteriosis in *Solea* *senegalensis***

**Marta A. Lages^1^, Miguel Balado^1,3*^, Abel M. Forero^2^, Lucía Ageitos^2^, Jaime Rodríguez^2^, Carlos Jiménez^2^, Manuel L. Lemos^1^, Beatriz Magariños^1*^**

^1^ Department of Microbiology and Parasitology, Aquatic One Health Research Center (ARCUS), University of Santiago de Compostela, 15782, Santiago de Compostela, Spain.

^2^ Department of Chemistry and CICA - Centro Interdisciplinar de Química e Bioloxía, University of A Coruña, 15071, A Coruña, Spain.

^3^ Department of Aquaculture and Blue Biotechnology, Centro Oceanográfico de Vigo (COV), Instituto Español de Oceanografía - Consejo Superior de Investigaciones Científicas (IEO-CSIC), 36390, Vigo, Spain.

* Corresponding authors: Miguel Balado (miguel.balado@csic.es); Beatriz Magariños (beatriz.magarinos@usc.es)

Index

[Fig. S1: Schematic representation of rFrpA encapsulation into liposomes.	2](#_Toc222394736)

[Fig. S2. SDS-PAGE and Western blot analysis of purified rFrpA following Ni²⁺ affinity chromatography	3](#_Toc222394737)

[Fig. S3. Ultrastructural characterization of rFrpA-loaded liposomes.	4](#_Toc222394738)

Figure S1. Schematic representation of rFrpA encapsulation into liposomes by the hydration–extrusion method. Lipid films composed of 1-palmitoyl-2-oleoyl-sn-glycero-3-phosphocholine (POPC) were hydrated with rFrpA in PBS and extruded through polycarbonate membranes to generate unilamellar proteoliposomes.

B

A

MW

kDa

**←**

75→

63→

**←**

Figure S2. SDS-PAGE and Western blot analysis of purified recombinant FrpA following Ni²⁺ affinity chromatography. (A) SDS-PAGE image of fractions containing rFrpA obtained after purification using an FPLC system (Bio-Rad NGC) equipped with a 1 mL HisTrap nickel affinity column (Cytiva). (B) Western blot analysis of the rFrpA samples used for vaccine formulation. Arrows indicate the band corresponding to rFrpA detected at the expected molecular mass (≈70 kDa).

Figure S3. Ultrastructural characterization of rFrpA-loaded liposomes. (A) TEM image showing spherical unilamellar vesicles (yellow arrows; scale bar = 200 nm). (B) SEM image showing spherical liposomes (scale bar = 1 µm).
